# Resistance to Experimental Visceral Leishmaniasis in Mice Infected With *Leishmania infantum* Requires Batf3

**DOI:** 10.3389/fimmu.2020.590934

**Published:** 2020-12-10

**Authors:** Manuel Soto, Laura Ramírez, José Carlos Solana, Emma C. L. Cook, Elena Hernández-García, Sara Charro-Zanca, Ana Redondo-Urzainqui, Rosa M. Reguera, Rafael Balaña-Fouce, Salvador Iborra

**Affiliations:** ^1^ Centro de Biología Molecular Severo Ochoa (CSIC-UAM), Departamento de Biología Molecular, Nicolás Cabrera 1, Universidad Autónoma de Madrid, Madrid, Spain; ^2^ Department of Immunology, Ophthalmology and ENT, School of Medicine, Universidad Complutense de Madrid, Madrid, Spain; ^3^ Departamento de Ciencias Biomédicas, Universidad de León, León, Spain

**Keywords:** *Leishmania*, Batf3, visceral leishmaniasis, dendritic cells, Batf3 DC+, Th1 responses

## Abstract

Unveiling the protective immune response to visceral leishmaniasis is critical for a rational design of vaccines aimed at reducing the impact caused by this fatal, if left untreated, vector-borne disease. In this study we sought to determine the role of the basic leucine zipper transcription factor ATF-like 3 (Batf3) in the evolution of infection with *Leishmania infantum*, the causative agent of human visceral leishmaniasis in the Mediterranean Basin and Latin America. For that, Batf3-deficient mice in C57BL/6 background were infected with an *L. infantum* strain expressing the luciferase gene. Bioluminescent imaging, as well as *in vitro* parasite titration, demonstrated that Batf3-deficient mice were unable to control hepatic parasitosis as opposed to wild-type C57BL/6 mice. The impaired microbicide capacities of *L. infantum*-infected macrophages from Batf3-deficient mice mainly correlated with a reduction of parasite-specific IFN-γ production. Our results reinforce the implication of Batf3 in the generation of type 1 immunity against infectious diseases.

## Introduction

Human leishmaniases are a group of poverty-related neglected diseases caused by an infection with parasites of the genus *Leishmania*, which are transmitted by infected sand flies. Depending on the infectious parasitic species, patients may develop different pathologies. In the Old World, cutaneous leishmaniasis (CL) is mainly caused by *L. major* infection. Visceral leishmaniasis (VL) usually develops after infection with *L. infantum* (Mediterranean countries and South America) or *L. donovani* (Indian Subcontinent and East Africa) (1). Even though patients who have recovered from leishmaniasis develop immunity against reinfection (2), suggesting that an effective vaccine should be feasible, to date there are no vaccines or specific immunotherapies against the human forms of the disease.

The existence of murine models of infection for different parasitic species is contributing to the development of vaccines or more effective and advanced therapeutic strategies (3, 4). In addition, these models have been fundamental to understand the generation, maintenance and, eventually, failure of those immune responses underlying either resistance or susceptibility to infection. Different studies performed on mice experimentally infected with *L. major* have allowed the establishment of an association between resistance and susceptibility, and the cellular response induced after challenge. The susceptibility to infection shown by BALB/c mice correlates with the induction of a dominant Interleukin (IL)-4–producing CD4^+^ Th2 response, and to the generation of parasite-dependent IL-10 responses (5). As a result, the parasite multiplies in the site of infection and subsequently spreads to the viscera (6). Alternatively, resistant C57BL/6 mice develop a response mediated by Interferon (IFN)-γ-producing CD4 ^+^ Th1 cells, thus activating infected macrophages to produce nitric oxide (NO), which mediates the intracellular killing of the parasite (5).

Infection of both BALB/c or C57BL/6 strains with *Leishmania* viscerotropic species results in parasite multiplication in the liver, spleen and bone marrow (6). During the first weeks after challenge (initial phase) parasites multiply in the liver, but in the late phase infected Kupffer cells are activated to produce NO resulting in a decrease of hepatic parasitic burdens. This inflammatory response is unable to control parasite multiplication in either the spleen or the bone marrow, resulting in a chronic infection (7, 8). The immune response concomitant to this parasitosis evolution after challenge with *L. infantum* has been mainly studied in the BALB/c model and results in the generation of both Th1 and Th2 responses (9–11). This mixed Th1/Th2 response has been also recently reported in the C57BL/6-*L. infantum* model of infection (12).

AP-1 (activator protein-1) constitutes a family of transcription factors endowed with a basic region-leucine zipper (bZIP) belonging to different families (JUN, FOS, ATF, basic leucine zipper transcriptional factor ATF-like and MAF) that form heterodimers to regulate transcription. Development and function of myeloid and lymphoid cell populations is regulated by different basic leucine zipper transcriptional factor ATF-like (BATF) proteins (13). BATF proteins can act as negative regulators of the AP-1 complex or interact with IFN regulatory factor (IRF) family member to regulate transcription (14). Batf3 is a BATF member that was firstly identified in human T cells and is required for the development of the of a subset of conventional dendritic cells (DC) (15). To determine the role of Batf3 in visceral leishmaniasis (VL), we have employed C57BL/6 wild-type and *Batf3* ^−/−^ animals challenged with an infective genetically-modified strain expressing red-shifted luciferase (luc) gene (16). We have followed the presence of parasites in internal organs *in vivo* throughout the course of the infection. We have analyzed the presence of viable parasites in liver, spleen and bone marrow in the initial (fourth week) and late phases (tenth week). We have also examined the immune response in animals in both phases, studying the humoral and cellular responses against the parasite. We found an enhanced susceptibility along with a decrease in the parasite-specific CD4^+^ Th1 response in *L. infantum*-infected Batf3-deficient mice, while the proportions of IL-4, IL-17, or IL-10–producing CD4^+^ T cells remained unaffected. These results suggest the involvement of Batf3-dependent conventional type 1 DC (cDC1) in resistance to VL, but further studies will be required to directly demonstrate their role in the susceptibility to this infectious disease. Thus, we show that, although the absence of Batf3 critically impairs Th1 immunity, it might have a differential impact on other T cell subsets or not, depending on the pathogen.

## Materials and Methods

### Mice and Parasites

Mice were bred under specific pathogen-free conditions at the National Centre for Cardiovascular Research (CNIC) and transported to the Severo Ochoa Molecular Biology Centre (CBMSO) to conduct the research. *Batf3* ^−/−^ mice kindly provided by Dr. Kenneth M. Murphy, (Washington University, St. Louis, MO. USA), were backcrossed more than ten times to establish WT and *Batf3* ^−/−^colonies from the heterozygotes. The animal research complies with EU Directive 2010/63EU, Recommendation 2007/526/EC and Spanish Royal Decree (RD 53/2013) regarding the protection of animals used for experimental and other scientific purpose. Procedures were approved by the Animal Care and Use Committee at the Centro de Biología Molecular Severo Ochoa (CEEA-CBMSO 23/243), the Bioethical Committee of the CSIC (under reference 795/2019), Bioethical Committees of the CNIC and Universidad Complutense de Madrid as well as the Government of the Autonomous Community of Madrid (Spain) under the references PROEX 115/19, PROEX 121/14, and PROEX134/19. All animal procedures conformed to EU Directive 2010/63EU and Recommendation 2007/526/EC regarding the protection of animals used for experimental and other scientific purposes, enforced in Spanish law under Real Decreto 1201/2005. All experimentation was performed with female mice.

The *PpyRE9h* **^+^** *L. infantum* strain expressing red-shifted luc gene was employed to infect the mice (16). Promastigotes were cultured at 26°C in M3 medium supplemented with 10% fetal calf serum (FCS; Sigma. St. Louis MO. USA), 100 U/ml of penicillin, 100 μg/ml of streptomycin and 100 μg/ml of puromycin (all purchased from Thermo Fischer Scientific, Waltham, MA, USA). Animals were challenged intravenously (i.v.) with 1 × 10^7^ stationary phase promastigotes. For soluble leishmania antigen (SLA) preparation, *L. infantum* (strain MCAN/ES/96/BCN150) promastigotes were employed. This strain was grown in the same medium indicated above but in the absence of puromycin.

### Follow-Up of *In Vivo* Infections by Bioluminescent Imaging and *In Vitro *Quantification in Internal Organs of the Parasitic Burdens

After challenging the mice with *PpyREh9+L. infantum* promastigotes, animals were monitored weekly in a Charge-Coupled Device (CCD) IVIS 100 Xenogen system (Caliper Life Science, Hopkinton, MA, USA) as described in (16). Briefly, images were acquired for 10 min from animals anesthetized with isoflurane that were previously intraperitoneally injected with D-luciferin (150 mg/Kg) purchased from Perkin Elmer (Waltham, MA, USA). The estimation of the parasitic burden in living mice was performed by the quantification of the regions of interest (ROIs) around liver and femur in ventral position using Living Image v.4.3. The values of BLI are expressed as radiance (p/s/cm^2^/sr).

At the indicated time points post-challenge, parasitic burden in liver, spleen and bone marrow (BM) were determined by limiting dilution as described in (17). Briefly, approximately 20 mg of liver, the whole spleen, and BM samples perfused from the femur cavities of each mouse, were individually homogenized in M3 medium supplemented with 20% FCS, 100 U/ml of penicillin, 100 μg/ml of streptomycin and 100 μg/ml of puromycin and filtered through 70 μm cell strainers (Corning Gmbh, Kaiserslautern, Germany) to obtain a cell suspension. Cells were serially diluted (1/3) in 96-well flat-bottomed microtiter plates (Thermo Fischer Scientific) containing the same medium employed for homogenization (in triplicates). The number of viable parasites was determined from the highest dilution at which promastigotes could be observed after 10 days of incubation at 26°C and is indicated per whole spleen, per gram of liver, or parasites per 1 × 10^7^ BM cells.

### Analysis of the *Leishmania*-Specific Humoral Responses

The reactivity against parasite proteins was determined by ELISA as described in (18) using NUNC MaxySorp plates. Briefly, plate wells were coated with freeze-thawed *L. infantum* SLA (1.2 µg per well) and incubated with 1/2 dilutions (starting at 1/100). The serum of each infected mouse was analyzed independently. Anti-IgG1, or anti-IgG2c horseradish peroxidase-conjugated anti-mouse immunoglobulins from Nordic (BioSite Täby, Sweden) were used as secondary antibodies at 1/2,000 dilution. Orto-phenylenediamine was employed for color development and the optical densities were read at 490 nm in an ELISA microplate spectrophotometer (Model 680, Bio-Rad Laboratories). Sera reactivity was calculated as the reciprocal end-point titer defined as the inverse value of the highest serum dilution factor giving an absorbance > 0.1 absorbance unit.

### *In Vitro* Cell Stimulation, Determination of Cytokine Concentration in Culture Supernatants, and Analysis of IFN-γ Production by T Cells

Primary cultures from the spleen of infected mice were established in RPMI complete medium: RPMI medium (Sigma) supplemented with 10% heat-inactivated FCS, 20 mM L-glutamine, 200 U/ml penicillin, 100 μg/ml streptomycin and 50 μg/ml gentamicin (Thermo Fischer Scientific). GMCSF BM-derived CD11c^+^ cells (GM-BM) were used as antigen presenting cells. They were derived from BM suspensions cultured for 7 days in RPMI complete medium supplemented with 20 ng/ml recombinant GMCSF (Peprotech, London, UK). Cells were loaded with *L. infantum* SLA (3 µg/ml) the last 24 h to obtain stimulated cells. For *in vitro* stimulation, spleen cells (2 ×10^6^ cells/ml) were co-cultured at 37°C and 5% CO_2_ with GM-BM cells (4 ×10^5^ cell/ml) (stimulated or not with SLA) in RPMI complete medium.

For the analysis of cytokine secretion to culture supernatants, spleen cells were stimulated as indicated above for 72 h. Afterwards, supernatants were obtained and analyzed by sandwich ELISA using commercial kits (Thermo Fisher Scientific). The levels of IFN-γ, IL-17, IL-10, IL-4, or IL-13 in culture supernatants were determined.

For the analysis of cells producing IFN-γ, spleen cells were stimulated as indicated above for 48 h. For the last 6 h, cultures were treated with brefeldin A (10 µg/ml; Sigma). Then, cells were harvested, washed in PBS with 1% heat-inactivated FCS (PBSw) and incubated with Fc block (BD Bioscience, San José, CA, USA) prior to staining. Next, cell surface markers: CD3 (clone 145-2C11; APC), CD4 (clone RM4-5; BV570) and CD8 (clone 53–6.7; FITC) were stained for 30 min at 4°C. After washing in PBSw, cells were fixed and permeabilized with Cytofix/Cytoperm (BD Bioscience). Next, the PE/Cy7 anti-mouse IFN-γ (clone XMG1.2) antibody was added for 30 min at 4°C. Finally, cells were washed and analyzed. Antibodies were purchased from BioLegend (San Diego, CA, USA). Samples were acquired using a FACS Canto II flow cytometer and FACSDiva Software (BD Bioscience) and processed and plotted with FlowJo Software (FlowJo LLC, Ashland, Oregon, USA).

### Nitrite Determination

Release of nitrite was determined in the supernatant of spleen cell cultures (5 × 10^6^ cell/ml) stimulated or not with Concanavalin A (ConA;1 µg/ml) or SLA (12 µg/ml) for 72 h in complete RPMI medium. For nitrite determination, 50 µl of culture supernatant were mixed with an equal volume of Griess reagent. Nitrite concentration was calculated from a sodium nitrite linear standard curve (1–100 µM). Absorbance was measured at 540 nm.

### Statistical Analysis

Statistical analysis was performed using the Graph-Pad Prism 5 program. Data were analyzed by a two-tailed Student *t*-test. Differences were considered significant when *P* < 0.05.

## Results

### Batf3 Deficiency Impacts on VL Progression in Mice Infected With *L. infantum*

**Figure 1:**
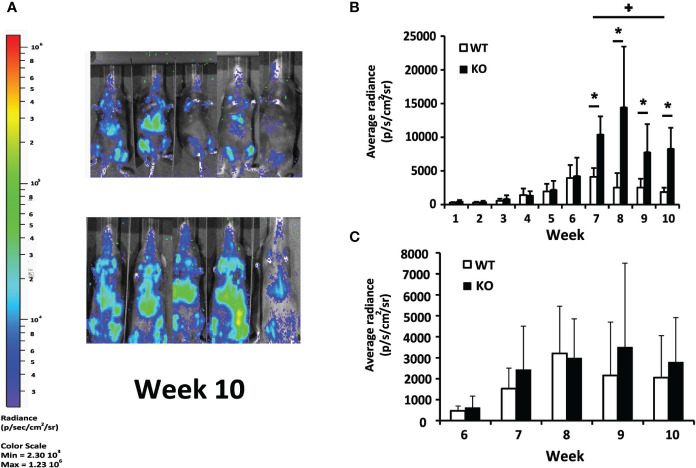
*In vivo* evaluation of *L. infantum* infection. *Batf3* ^−/−^ (KO; n= 9, weeks 1–3; n = 5, weeks 4–10) and wild-type (WT; n= 10, weeks 1–3; n = 5, weeks 4–10) mice were infected intravenously with 1 × 10^7^ PpyREh9+ *L. infantum* stationary phase promastigotes. **(A)** Representative bioluminescent images of infected mice. **(B, C)** The graphs show the mean (+ SD) of the quantification of ventral bioluminescence corresponding to the liver **(B)** or femurs **(C)** from the experiment shown in the **Supplementary Figure 1**. Symbol * shows the statistical differences between KO and WT mice data (P < 0.05). Symbol + shows the statistical differences between WT data taken at week 7 and at week 10 (P < 0.05). Results are representative of two independent experiments.

To analyze the role of Batf3 deficiency in the evolution of VL, we carried out a comparative analysis of the development of the infection in the liver with parasites expressing the luc gene between *Batf3* ^−/−^ mice and their corresponding littermate controls. No differences were observed in hepatic bioluminescence values between *Batf3* ^−/−^ and control mice at the initial phase of the infection (weeks 1–6; **Figure 1A** and **Supplementary Figure 1**). However, as can be deduced from the radiance values from week 7 to the end of the assay, at the late phase of the infection, parasite burden reached a plateau in *Batf3* ^−/−^ mice, suggesting that they have an impaired ability to control hepatic parasite multiplication (weeks 7–10; **Figures 1A, B** and **Supplementary Figure 1**). From week 6 onwards, similar parasite numbers were detected in the bone marrow of both groups (**Figure 1C** and **Supplementary Figure 1**). We could not detect parasites in the spleen with either the front view or the lateral view, except for some Batf3-deficient mice that, during the last two weeks, presented dispersion of parasites to other internal organs (**Supplementary Figure 1**).

**Figure 2:**
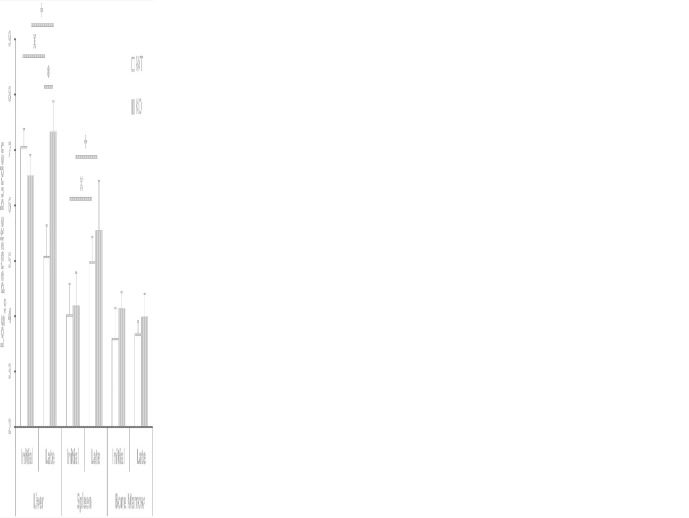
Evolution of parasite burden. The presence of viable parasites in *Batf3* ^−/−^ (KO) and wild-type (WT) mice infected intravenously with 1 × 10^7^ *PpyREh9^+^L. infantum* stationary phase promastigotes was determined at week 4 (initial; n= 4 KO, n = 5 WT) or at week 10 (late; n = 5 both groups) by limiting dilution. Data are represented as the mean (+ SD) of the parasite loads from each group. Samples from each mouse were independently determined in the liver (parasites per gr), spleen (parasites per organ) or bone marrow (parasites per 1 × 10^7^ cells). * (*P* < 0.05) shows the statistical differences in liver parasite loads from WT and KO groups. **^+^** (*P* < 0.05) shows the statistical differences in liver or spleens from mice of the KO group at week 4 and at week 10 post-challenge. **^x^** (*P* < 0.05) shows the statistical differences in liver or spleens from mice of the WT group at week 4 and at week 10 post-challenge.

Differences in hepatic parasitic burdens between *Batf3* ^−/−^ and control mice were confirmed by using a limiting dilution assay. *Batf3* ^−/−^ mice showed an increase of two orders of magnitude in hepatic parasitic load compared to controls at week 10 post-challenge. The ability to control parasite multiplication in the liver was only observed in wild-type mice, since there was a decrease in the number of parasites from the early to the late phase. However, mice deficient for Batf3 exhibit an increase in the number of parasites (**Figure 2**). We also determined the parasitic burden in the spleen. Although *Batf3* ^−/−^ mice presented higher parasite numbers than control mice in this organ, the increase was not found to be significantly different in any of the two repeat experiments. In both groups, we found splenic chronic progressive infection, since the number of parasites increased significantly in the spleen with infection time (**Figure 2**). Similar parasite numbers were found in the bone marrow from both groups at week 4 and week 10 post-challenge (**Figure 2**). All these results allowed us to conclude that the deficiency in Batf3 transcription factor prevents the control of parasite multiplication in the liver, without affecting the establishment of a chronic infection in the spleen or bone marrow. We found in some Batf3-deficient mice signs of illness around weeks 7 to 9 post-challenge: i.e. lethargy and unkempt coats, so we decided to establish a humane endpoint at 10 weeks to prevent unnecessary animal distress.

### Batf3 Deficiency Impairs the Generation of *Leishmania*-Specific IgG2c Humoral Response in Mice Infected With *L. infantum*

**Figure 3:**
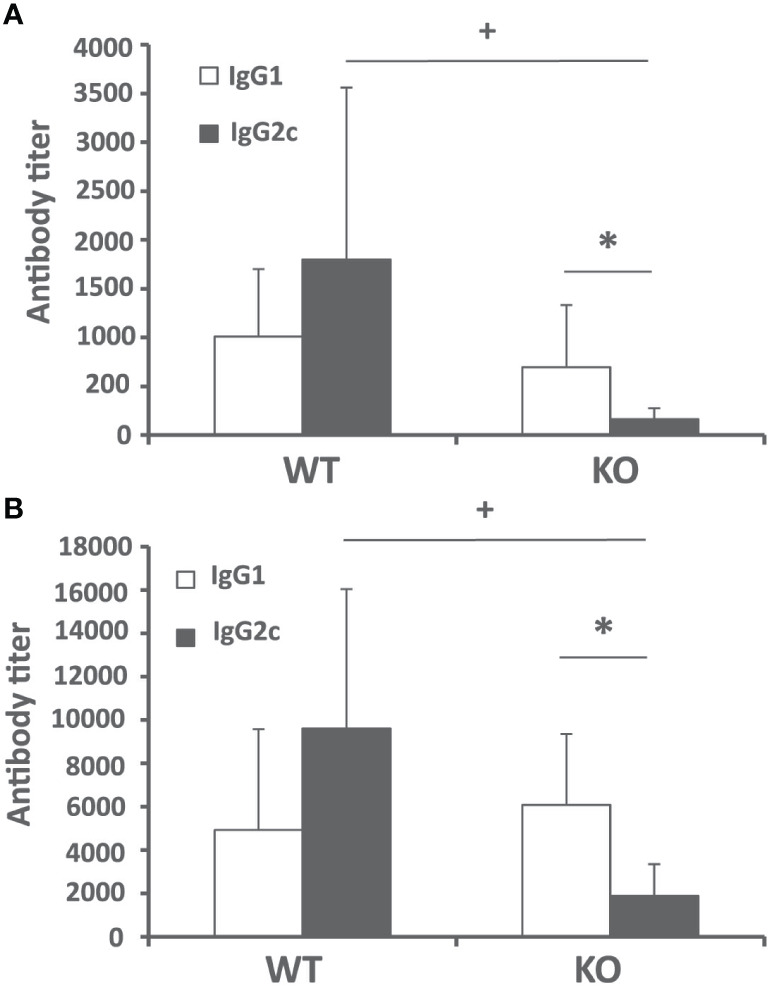
Humoral response against *Leishmania* after challenge. *Batf3* ^−/−^ (KO) and wild-type (WT) mice were infected intravenously with 1 × 10^7^ *PpyREh9* ^+^ *L. infantum* stationary phase promastigotes. The IgG1 and IgG2c reciprocal end-point titers against *L. infantum* SLA were calculated by ELISA at week 4 **(A)** (n = 9 KO, n = 10 WT) or week 10 **(B)** (n = 5 per group). Data are represented as mean + SD. * (*P* < 0.05) shows the statistical differences between IgG1 and IgG2c titers in the KO group. **^+^** (*P* < 0.05) shows the statistical differences of the IgG2c titers between WT and KO groups. Results are representative of two independent experiments.

Next, we analyzed the humoral immune response against leishmanial antigens in the *L. infantum* infected mice. We evaluated the titer of IgG2c and IgG1 anti-SLA antibodies in the sera of infected mice as an indication of the in vivo induction of Th1- or Th2-mediated responses respectively. The analysis was performed at week 4 after challenge (initial phase, **Figure 3A**) and at the end of the experiment 10 weeks after infection (late phase, **Figure 3B**). At both time points, mice of the control group showed a mixed response, with titers of IgG1 and IgG2c anti-SLA antibodies that were not significantly different. *Batf3* ^−/−^ mice showed very low, although detectable, titers of the IgG2c subclass antibodies throughout the infection process. These titers were significantly lower than those found in mice of the control group (**Figures 3A, B**). No differences were observed for the titer of anti-SLA IgG1 antibodies between both groups (**Figures 3A, B**). This diminished IgG2c response, which depends on IFN-γ (19), suggests that *Batf3* ^−/−^ mice have an impaired capacity to induce Th1 responses against the parasite that did not result in an increased parasite-specific Th2-mediated humoral response.

### Batf3 Deficiency Impairs the Generation of *Leishmania*-Specific Cellular Immune Responses in Mice Infected With *L. infantum*

**Figure 4:**
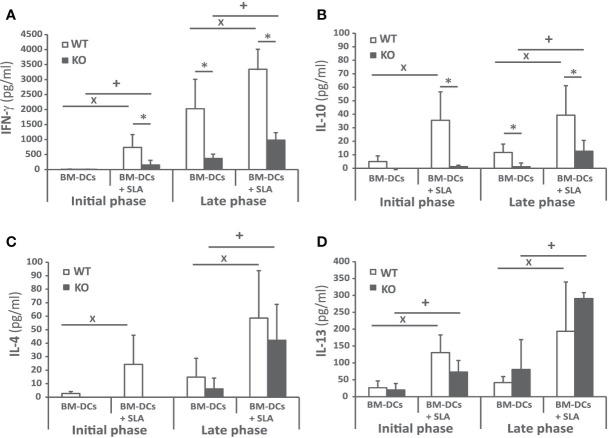
Cytokine production against the parasite after challenge. *Batf3* ^−/−^ (KO) and wild-type (WT) mice were infected intravenously with 1 × 10^7^ *PpyREh9* ^+^ *L. infantum* stationary phase promastigotes. Spleen cells cultures from each mouse were independently established at week 4 (Initial phase; n= 4 KO, n = 5 WT) or at week 10 (Late phase; n = 5 both groups) and stimulated for 72 h with BM-DCs pulsed or not with SLA. IFN-γ **(A)**, IL-10 **(B)**, IL-4 **(C)**, or IL-13 **(D)** levels were measured in culture supernatants by quantitative sandwich ELISA. Data are represented as the mean + SD. * (*P* < 0.05) shows the statistical differences between WT and KO mice groups. **^+^** (*P* < 0.05) shows the statistical differences between the SLA stimulated and unstimulated cells from mice of the KO group. **^x^** (*P* < 0.05) shows the statistical differences between the SLA stimulated and unstimulated cells from mice of the WT group. Results are representative of two independent experiments (Late phase).

To investigate the cellular immune response elicited against the parasite, we analyzed *Leishmania*-specific cytokine secretion by spleen cells taken at week 4 and at week 10 post-infection ex vivo. For stimulation, we used CD11c^+^ GMCSF bone marrow-derived cells (GM-BM) loaded or not with *L. infantum* SLA were employed. The SLA-specific production of IFN-γ was higher in wild-type animals at the initial and at the late phase of infection, (**Figure 4A**). A similar profile was obtained when determining the presence of IL-10 in culture supernatants (**Figure 4B**). The only difference was the absence of IL-10 in the supernatant of the cultures established from the *Batf3* ^−/−^ mice independently of the stimuli at the initial phase (**Figure 4B**). Regarding Th2-related cytokines, SLA-dependent secretion of IL-4 (**Figure 4C**) and IL-13 (**Figure 4D**) was observed at the late phase for both cytokines and also at the initial phase for IL-13. At the late phase of the infection we did not detect significant differences between the amount of these cytokines in both mice groups. We did not detect a SLA-dependent secretion of IL-17 in any group (**Supplementary Figure 2**).

**Figure 5:**
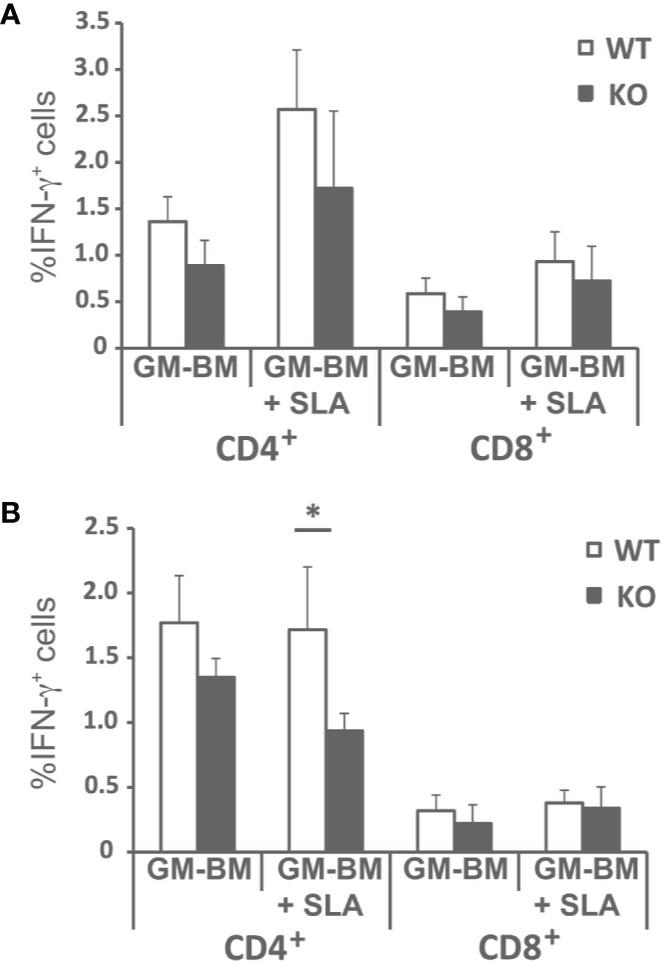
Percentages of T cell producing IFN-γ. *Batf3* ^−/−^ (KO) and wild-type (WT) mice were infected intravenously with 1 × 10^7^ PpyREh9^+^ *L. infantum* stationary phase promastigotes. Spleen cells cultures from each mouse were independently established at week 4 (**A**; Initial phase; n= 4 KO, n = 5 WT) or at week 10 (**B**; Late phase; n = 5 both groups) and stimulated for 48 h with BM-DCs pulsed or not with SLA. Afterwards cells were processed for flow cytometry. Analyses are gated on CD3^+^ cells. Data are represented as the mean (+ SD) of the percentage of CD4^+^ or CD8^+^ T cells positive for IFN-γ. * (*P* < 0.05) shows the statistical differences between WT and KO mice groups.

Since the predominant IFN-γ-mediated response found in the infected wild-type animals was decreased, although not absent, in *Batf3* ^−/−^ mice, we analyzed the frequency of CD4^+^ and CD8^+^ T cells producing this cytokine in both mice groups. **Supplementary Figure 3** shows the gating strategies and FMO controls. The frequency of IFN-γ-producing CD4^+^ and CD8^+^ T cells was analyzed both at the initial (**Figure 5A**), and at the late phase of the infection (**Figure 5B**). We detected a statistically significant higher production of FN-γ-producing only for CD4^+^ T cells stimulated with SLA at the final phase of the infection.

**Figure 6:**
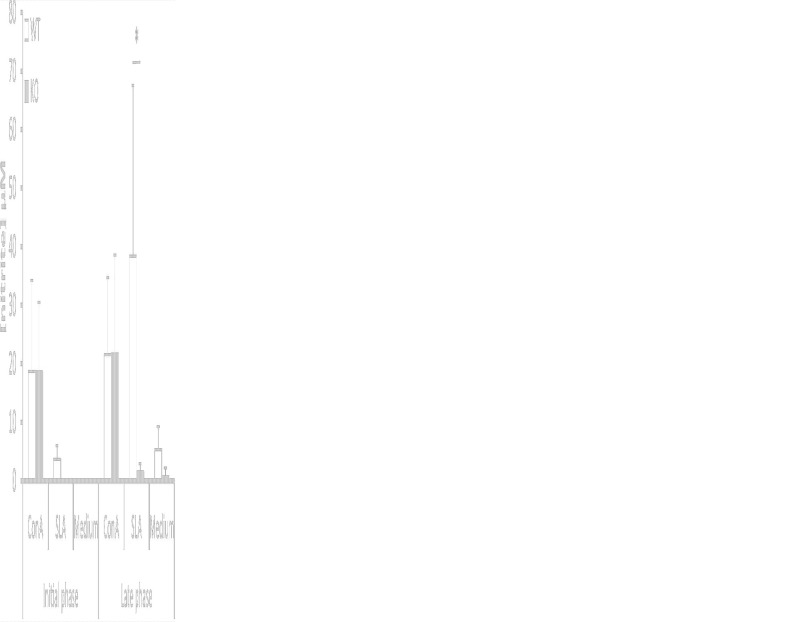
Determination of the amounts of nitrite in the supernatants of splenic cell cultures. *Batf3* ^−/−^ (KO) and wild-type (WT) mice were infected intravenously with 1 × 10^7^ PpyREh9^+^ *L. infantum* stationary phase promastigotes. Spleen cells cultures from each mouse were independently established at week 4 (Initial phase; n= 4 KO, n = 5 WT) or at week 10 (Late phase; n = 5 both groups) and grown for 72 h without stimulus (Medium), treated with ConA or pulsed with SLA. Mean (+ SD) of nitrite levels is shown. * (P < 0.05) shows the statistical differences between WT and KO mice groups. Data shown are representative of two independent experiments with similar results (Late phase).

To complement these findings, we measured the amounts of nitrites, derived from NO production in macrophages, by Griess reaction in the culture supernatants of spleen cells established from *Batf3* ^−/−^ and control mice. Our results showed the presence of nitrites in cell supernatants from WT mice upon stimulation with SLA, in greater magnitude in the late phase of infection (**Figure 6**). This metabolite was almost absent in cultures generated from Batf3-deficient mice stimulated with SLA, but produced in similar levels than in WT mice upon Concanavalin A treatment, reflecting the limited leishmanicidal capacity of their macrophages in response to parasite stimuli.

## Discussion

Batf3 transcription factor may play different roles in the development and function of myeloid and lymphoid populations. For example, it inhibits the differentiation of regulatory T cells in the periphery (20). Very recently, a role for Batf3 in memory CD8^+^ T cell differentiation has been revealed (21, 22). Importantly, Batf3 selectively determines acquisition of CD8α^+^ DC phenotype and function in lymphoid organs, and CD103^+^ in non-lymphoid organs (15, 23–25). Both subsets comprise conventional type 1 DCs (cDC1), which play important roles in viral and cancer immunity (26).

The chronic self-healing *L. major* infection model in C57BL/6 mice contributed to unveiling the role of the different DC subsets in the priming and maintenance of the anti-*Leishmania* response [reviewed in (27, 28)]. DC comprise a heterogenous population, and are further classified into distinct subtypes according to ontogeny, differential expression of surface proteins, cell localization, and immunological function. Intradermal infection of a C57BL/6 mouse strain deficient in Batf3, resulted in an exacerbated evolution of the CL disease (29, 30). In this model, it was demonstrated that Batf3-dependent DCs are essential for the control of CL due to *L. major* infection. Batf3-deficient mice presented unresolved lesions and higher parasitic burdens, because of their inability to produce IL-12, a cytokine which is critical for the maintenance of Th1 immunity (29). In contrast to the mouse model of *L. major*-induced CL, less is known about the role of the different DC subsets in VL. We hypothesized that absence of cDC1, due to Batf3-deficiency, might impact on cross-priming of IFN-γ CD8^+^ T cells (15, 31, 32), which play a major role in experimental VL (32, 33) and/or maintenance of IFN-γ-producing CD4^+^ T cell responses (29, 35).

Murine models of VL have been extensively employed to elucidate the interaction of the host's immune system and viscerotropic parasite species (36, 37). Using *in vivo* bioimaging techniques we have been able to follow the evolution of the parasite colonization in C57BL/6 mice infected by *L. infantum*. According to previous reports (16, 38, 39) we have employed the pseudocolor heat-map signals to analyze the evolution of the parasitic burden in liver, spleen and bone marrow (**Figure 1** and **Supplementary Figure 1**). We have also determined the parasite loads in these organs by titration assays at two time points corresponding to the initial phase, when the immune response is primed in the spleen, and to the late phase of infection, when hepatic resistance is observed and parasites are present chronically in the spleen and BM (**Figure 2**) (7, 40). Although C57BL/6-*L. infantum* is not the most frequent murine model of VL, the data collected in this work are roughly consistent with published reports (12). Data show a similar evolution of the disease in this model of VL to that occurring in BALB/c mice infected by *L. infantum* or *L. donovani*, or in C57BL/6 mice infected with *L. donovani*; namely if comparing the control of parasitic burdens in the liver and development of a chronic infection in the spleen or the bone marrow (7, 8, 11, 41). Regarding the evolution of the hepatic parasite burden, bioluminescence reached its peak in weeks 6 to 7 and showed a progressive decline from those weeks until the end of the trial in wild-type mice. Interestingly, deficiency in Batf3 protein altered this evolution of hepatic parasitic load, which was progressively increased in *Batf3* ^−/−^ mice resulting in a chronic hepatic infection. It is noteworthy that in the last two weeks a dispersion of bioluminescence to other internal organs was observed in some *Batf3* ^−/−^ deficient mice, thus demonstrating a lack of control of the disease in Batf3-deficient animals. Contrary to the data shown by Alvarez et al. where they use the same *Leishmania* strain for infection of BALB/c mice (16), we were not able to detect bioluminescent signal in the spleen (except in some of the *Batf3* ^−/−^ animals at the end of the assays, as depicted above). Interestingly, a positive signal was obtained in the bone marrow (of the femur) in both animal groups. The higher concentration of parasites in the bone marrow is likely to allow their visualization, while in the spleen the luminescence remains below the detection threshold. The lower parasitic load observed in the spleen of C57BL/6 mice compared to BALB/c animals infected with the same *L. infantum* strain (10, 16) is consistent with previous data indicating that, although both strains are considered susceptible to VL, C57BL/6 mice show an intermediate phenotype of infection, less susceptible than BALB/c mice (2, 12, 42, 43). However, and as reported previously for C57BL/6 infected with *L. infantum* (12) or *L. donovani* (42), an increase of splenic parasite burdens with time was observed in both wild-type and *Batf3* ^−/−^ animals.

The self-resolving liver infection of the murine VL models mainly relies on the development of a CD4^+^ Th1 response specific for the parasite that is primed in the spleen and is implicated in the formation of inflammatory granulomas in the liver (40). The activation of this CD4^+^ Th1 cell response mounted by DCs occurs in the spleen in a IL-12–dependent manner (7, 11, 37). Here, we present different evidence about the existence of a dampened inflammatory response in *Batf3* ^−/−^ mice both at the initial and late phases of the disease compared to wild-type mice. The first evidence is the limited presence of anti-SLA IgG2c subclass antibodies in the serum of knockout mice at both phases of infection. Thus, we found significantly reduced titers of this antibody subclass in *Batf3* ^−/−^ animals compared to wild-type mice (**Figures 3A, B**). The second evidence is related to the observation that *Batf3* ^−/−^ mice exhibit reduced production of parasite-specific IFN-γ by CD4^+^ T cells. This cytokine, that is essential for the activation of infected macrophages (37, 44), was found at significantly lower levels in the supernatants of cultures established from Batf3-deficient mice than in those of wild-type controls (**Figure 4A**). This finding correlates to the low NO production found in Batf3^−/−^ mice (**Figure 6**). Thus, Batf3-deficiency resulted in a decrease in global leishmanicidal capacity, since NO promotes the destruction of parasites that infect macrophages (45). Others and we (29, 30) have shown that, in the absence of Batf3-depedendent cDC1, there is a poor IFN-γ response in the murine model of CL due to infection with *L. major*. This highlights the involvement of Batf3 in the generation of Th1 immunity against different *Leishmania* species, as also happens with other protozoan parasites such as *Toxoplasma gondii* (35), or in mucosal or systemic bacterial infections (46). Although there is evidence indicating that CD8^+^ T cells can also contribute to the production of IFN-γ in murine models of VL (47, 48), and that Batf3-dependent DC are critical for CD8^+^ T cell crosspriming (15, 31, 32), we did not detect statistically significant differences in the percentages of CD8^+^ T cells producing IFN-γ between wild-type and Batf3-deficient animals (**Figures 5A, B**). We have not detected the generation of Th17 responses against *L. infantum* proteins (**Supplementary Figure 2**). This result contrasts with what has previously been reported for the CL murine model in *Batf3* ^−/−^ mice, where the defect in Th1 response was compensated by the induction of parasite-dependent IL-17–mediated responses (30).

Contrary to what occurs upon *L. major* infection, the negative effect of the absence of Batf3-dependent DC in the generation of Th1 responses does not correlate with an increase in Th2-type responses (29, 30). Thus, we show that parasite-specific IL-4 (**Figure 4C**) and IL-13 production (**Figure 4D**) and the titers of SLA-specific IgG1 circulating antibodies (**Figures 3A, B**) did not differ between the two mice groups, which could be related with the paucity of splenic Tfh cells observed in *L. infantum* C57BL/6 mice (12). Finally, IL-10 has been described as an immunoregulatory molecule in human and murine experimental VL that can suppress antiparasitic immunity but also contributes to tissue damage prevention (49–51). As occurred for Th2-related cytokines, the absence of Batf3 resulted in an increased production of IL-10 after *L. major* infection (28, 29), that is not reproduced in the VL model employed for this work (**Figure 4B**). Since IL-10 production by CD4^+^ Th1 cells could act as a feedback control of IFN-γ-mediated inflammation (51–53), the decreased production of IL-10 detected in Batf3^−/−^ mice splenocytes could be an indirect effect of the diminished IFN-γ responses observed in these animals.

In summary, our results suggest that Batf3-dependent DCs are essential for controlling VL induced by *L. infantum* infection. Notwithstanding, our results imply, but do not directly demonstrate the role of cDC1 in resistance to VL, and further studies are needed to address this important limitation. In the absence of this population, the induction of CD4^+^ Th1 responses against the viscerotropic *L. infantum* species is severely impaired, as has been described for CL. In contrast to *L. major*-infected Batf3-deficient mice, enhanced susceptibility to VL did not correlate with increased production of IL-10, Th2-type (29) or Th17-mediated responses (30). Thus, the crucial role of Batf3-dependent DCs, differentially imprinting Th responses in the different forms of leishmaniasis, reinforces the possibility of designing vaccines or targeted immunotherapies based on delivery of parasite antigens to this type of DCs.

## Data Availability Statement

The raw data supporting the conclusions of this article will be made available by the authors, without undue reservation.

## Ethics Statement

The animal study was reviewed and approved by Animal Care and Use Committee at the Centro de Biología Molecular Severo Ochoa (CEEA-CBMSO 23/243), the Bioethical Committee of the CSIC (under reference 795/2019) Bioethical Committees of the CNIC and Universidad Complutense de Madrid as well as the Government of the Autonomous Community of Madrid (Spain) under the references PROEX 115/19, PROEX 121/14 and PROEX134/19.

## Author Contributions

Conceptualization: MS and SI. Methodology: MS, SI, LR, JS, and RR. Investigation, MS, LR, JS, SC-Z, AR-U, EH-G, EC, and SI. Writing—Original Draft: MS and SI. Writing—Review and Editing: MS, LR, JS, SC-Z, AR-U, EH-G, EC, and SI. Funding Acquisition: MS and SI. Resources: RR and RB-F. Supervision: MS and SI. All authors contributed to the article and approved the submitted version.

## Funding

The research made for this study was supported in Spain by grants from Ministerio de Ciencia e Innovación FISPI11/00095 and FISPI14/00366 (FEDER FUNDING) and the Fondo de Investigaciones Sanitarias (ISCIII-RETICRD16/0027/0008-FEDER). SI is funded by RYC-2016-19463 and RTI2018-343 094484-B-I00 from Ministerio de Ciencia e Innovación (FEDER FUNDING). Institutional grants from the Fundación Ramón Areces and Banco de Santander to the CBMSO are also acknowledged. The funders had no role in study design, data collection and analysis, decision to publish, or preparation of the manuscript.

## Conflict of Interest

The authors declare that the research was conducted in the absence of any commercial or financial relationships that could be construed as a potential conflict of interest.

## References

[1] Steverding D The history of leishmaniasis. Parasit Vectors (2017) 10:82. 10.1186/s13071-017-2028-5 28202044 PMC5312593

[2] Gillespie PM Beaumier CM Strych U Hayward T Hotez PJ Bottazzi ME Status of vaccine research and development of vaccines for leishmaniasis. Vaccine (2016) 34:2992–5. 10.1016/j.vaccine.2015.12.071 26973063

[3] Uliana SRB Trinconi CT Coelho AC Chemotherapy of leishmaniasis: present challenges. Parasitology (2018) 145:464–80. 10.1017/S0031182016002523 28103966

[4] Iborra S Solana JC Requena JM Soto M Vaccine candidates against *Leishmania* under current research. Expert Rev Vaccines (2018) 17(4):323–34. 10.1080/14760584.2018.1459191 29589966

[5] Scott P Novais FO Cutaneous leishmaniasis: immune responses in protection and pathogenesis. Nat Rev Immunol (2016) 16:581–92. 10.1038/nri.2016.72 27424773

[6] Sacks DL Melby PC Animal models for the analysis of immune responses to leishmaniasis. Curr Protoc Immunol (2015) 108 19(2):1–24. 10.1002/0471142735.im1902s108 25640990

[7] Engwerda CR Kaye PM Organ-specific immune responses associated with infectious disease. Immunol Today (2000) 21:73–8. 10.1016/S0167-5699(99)01549-2 10652464

[8] Loeuillet C Banuls AL Hide M Study of *Leishmania* pathogenesis in mice: experimental considerations. Parasit Vectors (2016) 9:144. 10.1186/s13071-016-1413-9 26969511 PMC4788862

[9] Rolao N Cortes S Gomes-Pereira S Campino L *Leishmania infantum*: mixed T-helper-1/T-helper-2 immune response in experimentally infected BALB/c mice. Exp Parasitol (2007) 115:270–6. 10.1016/j.exppara.2006.09.013 17087930

[10] Carrion J Nieto A Iborra S Iniesta V Soto M Folgueira C Immunohistological features of visceral leishmaniasis in BALB/c mice. Parasite Immunol (2006) 28:173–83. 10.1111/j.1365-3024.2006.00817.x 16629702

[11] Nieto A Dominguez-Bernal G Orden JA De La Fuente R Madrid-Elena N Carrion J Mechanisms of resistance and susceptibility to experimental visceral leishmaniosis: BALB/c mouse versus syrian hamster model. Vet Res (2011) 42:39. 10.1186/1297-9716-42-39 21345200 PMC3052183

[12] Perez-Cabezas B Cecilio P Gaspar TB Gartner F Vasconcellos R Cordeiro-da-Silva A Understanding Resistance vs. Susceptibility in Visceral Leishmaniasis Using Mouse Models of *Leishmania infantum* Infection. Front Cell Infect Microbiol (2019) 9:30. 10.3389/fcimb.2019.00030 30881923 PMC6407322

[13] Murphy TL Tussiwand R Murphy KM Specificity through cooperation: BATF-IRF interactions control immune-regulatory networks. Nat Rev Immunol (2013) 13:499–509. 10.1038/nri3470 23787991

[14] Li P Spolski R Liao W Wang L Murphy TL Murphy KM BATF-JUN is critical for IRF4-mediated transcription in T cells. Nature (2012) 490:543–6. 10.1038/nature11530 PMC3537508 22992523

[15] Hildner K Edelson BT Purtha WE Diamond M Matsushita H Kohyama M Batf3 deficiency reveals a critical role for CD8alpha+ dendritic cells in cytotoxic T cell immunity. Science (2008) 322:1097–100. 10.1126/science.1164206 PMC2756611 19008445

[16] Alvarez-Velilla R Gutierrez-Corbo MDC Punzon C Perez-Pertejo MY Balana-Fouce R Fresno M A chronic bioluminescent model of experimental visceral leishmaniasis for accelerating drug discovery. PLoS Negl Trop Dis (2019) 13:e0007133. 10.1371/journal.pntd.0007133 30763330 PMC6392311

[17] Buffet PA Sulahian A Garin YJ Nassar N Derouin F Culture microtitration: a sensitive method for quantifying *Leishmania infantum* in tissues of infected mice. Antimicrob Agents Chemother (1995) 39:2167–8. 10.1128/AAC.39.9.2167 PMC162906 8540741

[18] Solana JC Ramirez L Corvo L de Oliveira CI Barral-Netto M Requena JM Vaccination with a *Leishmania infantum* HSP70-II null mutant confers long-term protective immunity against *Leishmania major* infection in two mice models. PLoS Negl Trop Dis (2017) 11:e0005644. 10.1371/journal.pntd.0005644 28558043 PMC5466331

[19] Gracie JA Bradley JA Interleukin-12 induces interferon-gamma-dependent switching of IgG alloantibody subclass. Eur J Immunol (1996) 26:1217–21. 10.1002/eji.1830260605 8647195

[20] Lee W Kim HS Hwang SS Lee GR The transcription factor Batf3 inhibits the differentiation of regulatory T cells in the periphery. Exp Mol Med (2017) 49:e393. 10.1038/emm.2017.157 29147008 PMC5704186

[21] Qiu Z Khairallah C Romanov G Sheridan BS Cutting Edge: Batf3 Expression by CD8 T Cells Critically Regulates the Development of Memory Populations. J Immunol (2020) 205:901–6. 10.4049/jimmunol.2000228 PMC7539233 32669309

[22] Ataide MA Komander K Knopper K Peters AE Wu H Eickhoff S BATF3 programs CD8(+) T cell memory. Nat Immunol (2020) 21:1397–407. 10.1038/s41590-020-0786-2 32989328

[23] Edelson BT Bradstreet TR Wumesh KC Hildner K Herzog JW Sim J Batf3-dependent CD11b(low/-) peripheral dendritic cells are GM-CSF-independent and are not required for Th cell priming after subcutaneous immunization. PLoS One (2011) 6:e25660. 10.1371/journal.pone.0025660 22065991 PMC3196467

[24] Seillet C Jackson JT Markey KA Brady HJM Hill GR MacDonald KPA CD8α+ DCs can be induced in the absence of transcription factors Id2, Nfil3, and Batf3. Blood (2013) 121:1574–83. 10.1182/blood-2012-07-445650 23297132

[25] Waithman J Zanker D Xiao K Oveissi S Wylie B Ng R Resident CD8(+) and Migratory CD103(+) Dendritic Cells Control CD8 T Cell Immunity during Acute Influenza Infection. PLoS One (2013) 8:e66136. 10.1371/journal.pone.0066136 23750278 PMC3672151

[26] Böttcher JP Reis e Sousa CR The Role of Type 1 Conventional Dendritic Cells in Cancer Immunity. Trends Cancer (2018) 4(11):784–92. 10.1016/j.trecan.2018.09.001 10.1016/j.trecan.2018.09.001 PMC6207145 30352680

[27] von Stebut E Tenzer S Cutaneous leishmaniasis: Distinct functions of dendritic cells and macrophages in the interaction of the host immune system with *Leishmania major* . Int J Med Microbiol (2017) 308:2206–214. 10.1016/j.ijmm.2017.11.002 29129568

[28] Martinez-Lopez M Soto M Iborra S Sancho D *Leishmania* Hijacks Myeloid Cells for Immune Escape. Front Microbiol (2018) 9:883. 10.3389/fmicb.2018.00883 29867798 PMC5949370

[29] Martinez-Lopez M Iborra S Conde-Garrosa R Sancho D Batf3-dependent CD103+ dendritic cells are major producers of IL-12 that drive local Th1 immunity against *Leishmania major* infection in mice. Eur J Immunol (2015) 45:119–29. 10.1002/eji.201444651 PMC4316187 25312824

[30] Ashok D Schuster S Ronet C Rosa M Mack V Lavanchy C Cross-presenting dendritic cells are required for control of *Leishmania major* infection. Eur J Immunol (2014) 44:1422–32. 10.1002/eji.201344242 24643576

[31] den Haan JM Lehar SM Bevan MJ CD8(+) but not CD8(-) dendritic cells cross-prime cytotoxic T cells in vivo. J Exp Med (2000) 192:1685–96. 10.1084/jem.192.12.1685 PMC2213493 11120766

[32] Theisen DJ Davidson JTT Briseno CG Gargaro M Lauron EJ Wang Q WDFY4 is required for cross-presentation in response to viral and tumor antigens. Science (2018) 362:694–9. 10.1126/science.aat5030 PMC6655551 30409884

[33] Polley R Stager S Prickett S Maroof A Zubairi S Smith DF Adoptive immunotherapy against experimental visceral leishmaniasis with CD8+ T cells requires the presence of cognate antigen. Infect Immun (2006) 74:773–6. 10.1128/IAI.74.1.773-776.2006 PMC1346645 16369038

[34] Stager S Rafati S CD8(+) T cells in leishmania infections: friends or foes? Front Immunol (2012) 3:5. 10.3389/fimmu.2012.00005 22566891 PMC3342007

[35] Mashayekhi M Sandau MM Dunay IR Frickel EM Khan A Goldszmid RS CD8alpha(+) dendritic cells are the critical source of interleukin-12 that controls acute infection by Toxoplasma gondii tachyzoites. Immunity (2011) 35:249–59. 10.1016/j.immuni.2011.08.008 PMC3171793 21867928

[36] Kaye PM Aebischer T Visceral leishmaniasis: immunology and prospects for a vaccine. Clin Microbiol Infect (2011) 17:1462–70. 10.1111/j.1469-0691.2011.03610.x 21851483

[37] Faleiro RJ Kumar R Hafner LM Engwerda CR Immune regulation during chronic visceral leishmaniasis. PLoS Negl Trop Dis (2014) 8:e2914. 10.1371/journal.pntd.0002914 25010815 PMC4091888

[38] Tavares J Costa DM Teixeira AR Cordeiro-da-Silva A Amino R In vivo imaging of pathogen homing to the host tissues. Methods (2017) 127:37–44. 10.1016/j.ymeth.2017.05.008 28522323

[39] Thalhofer CJ Graff JW Love-Homan L Hickerson SM Craft N Beverley SM In vivo imaging of transgenic *Leishmania* parasites in a live host. J Vis Exp (2010) (41):e1980. 10.3791/1980 PMC3156083 20689512

[40] Kaye PM Svensson M Ato M Maroof A Polley R Stager S The immunopathology of experimental visceral leishmaniasis. Immunol Rev (2004) 201:239–53. 10.1111/j.0105-2896.2004.00188.x 15361245

[41] Wilson ME Jeronimo SM Pearson RD Immunopathogenesis of infection with the visceralizing *Leishmania* species. Microb Pathog (2005) 38:147–60. 10.1016/j.micpath.2004.11.002 15797810

[42] Bodhale NP Pal S Kumar S Chattopadhyay D Saha B Chattopadhyay N Inbred mouse strains differentially susceptible to *Leishmania donovani* infection differ in their immune cell metabolism. Cytokine (2018) 112:12–5. 10.1016/j.cyto.2018.06.003 29885992

[43] Perez-Cabezas B Cecilio P Robalo AL Silvestre R Carrillo E Moreno J Interleukin-27 Early Impacts *Leishmania infantum* Infection in Mice and Correlates with Active Visceral Disease in Humans. Front Immunol (2016) 7:478. 10.3389/fimmu.2016.00478 27867384 PMC5095612

[44] Kaye P Scott P Leishmaniasis: complexity at the host-pathogen interface. Nat Rev Microbiol (2011) 9:604–15. 10.1038/nrmicro2608 21747391

[45] Olekhnovitch R Bousso P Induction, Propagation, and Activity of Host Nitric Oxide: Lessons from *Leishmania* Infection. Trends Parasitol (2015) 31:653–64. 10.1016/j.pt.2015.08.001 26440786

[46] Arnold IC Zhang X Artola-Boran M Fallegger A Sander P Johansen P BATF3-dependent dendritic cells drive both effector and regulatory T-cell responses in bacterially infected tissues. PLoS Pathog (2019) 15:e1007866. 10.1371/journal.ppat.1007866 31188899 PMC6590837

[47] Tsagozis P Karagouni E Dotsika E CD8(+) T cells with parasite-specific cytotoxic activity and a Tc1 profile of cytokine and chemokine secretion develop in experimental visceral leishmaniasis. Parasite Immunol (2003) 25:569–79. 10.1111/j.0141-9838.2004.00672.x 15053778

[48] Joshi T Rodriguez S Perovic V Cockburn IA Stager S B7-H1 blockade increases survival of dysfunctional CD8(+) T cells and confers protection against *Leishmania donovani* infections. PLoS Pathog (2009) 5:e1000431. 10.1371/journal.ppat.1000431 19436710 PMC2674929

[49] Bunn PT Montes de Oca M de Labastida Rivera F Kumar R Ng SS Edwards CL Distinct Roles for CD4(+) Foxp3(+) Regulatory T Cells and IL-10-Mediated Immunoregulatory Mechanisms during Experimental Visceral Leishmaniasis Caused by *Leishmania donovani* . J Immunol (2018) 201:3362–72. 10.4049/jimmunol.1701582 30355785

[50] Montes de Oca M Kumar R de Labastida Rivera F Amante FH Sheel M Faleiro RJ Blimp-1-Dependent IL-10 Production by Tr1 Cells Regulates TNF-Mediated Tissue Pathology. PLoS Pathog (2016) 12:e1005398. 10.1371/journal.ppat.1005398 26765224 PMC4713066

[51] Nylen S Sacks D Interleukin-10 and the pathogenesis of human visceral leishmaniasis. Trends Immunol (2007) 28:378–84. 10.1016/j.it.2007.07.004 17689290

[52] Stager S Maroof A Zubairi S Sanos SL Kopf M Kaye PM Distinct roles for IL-6 and IL-12p40 in mediating protection against *Leishmania donovani* and the expansion of IL-10+ CD4+ T cells. Eur J Immunol (2006) 36:1764–71. 10.1002/eji.200635937 PMC2659577 16791879

[53] Cope A Le Friec G Cardone J Kemper C The Th1 life cycle: molecular control of IFN-gamma to IL-10 switching. Trends Immunol (2011) 32:278–86. 10.1016/j.it.2011.03.010 21531623

