# Supplementary material for: Resistance to Experimental Visceral Leishmaniasis in Mice Infected With Leishmania infantum Requires Batf3
Source: Front Immunol. 2020 Dec 10;11:590934. doi: 10.3389/fimmu.2020.590934 (PMC7758202; doi:10.3389/fimmu.2020.590934)
Supplement: Supplementary file 1 [file DataSheet_1.pdf]

Supplementary material.

Supplementary Figure 1. Evaluation of *L. infantum* infection. Ventral view images of *Batf3*$^{-/-}$ (KO) and wild-type (WT) mice infected intravenously with 1×10^7 *PpyREh9*$^+$*L. infantum* stationary phase promastigotes. Pseudocolour heat-maps indicate intensity of bioluminescence from low (blue) to high (red). All images use the same scale heat-map (indicated at the right). Minimum and maximum radiance values are indicated. Images were taken at the indicated weeks post-challenge. Results are representative of two independent experiments.

Supplementary Figure 2. IL-17 production against the parasite after challenge. *Batf3*$^{-/-}$ (KO) and wild-type (WT) mice were infected intravenously with 1×10^7 *PpyREh9*$^+$ *L. infantum* stationary phase promastigotes. Spleen cells cultures from each mouse were independently established at week 4 (Initial phase; n= 4 KO, n = 5 WT) or at week 10 (Late phase; n = 5 both groups) and stimulated for 72 h with BM-DCs pulsed or not with SLA. IL-17 levels were measured in culture supernatants by quantitative sandwich ELISA. Data are represented as the mean + SD. No statistically differences were found.

Supplementary Figure 3. Analysis of splenic T cell populations. (A) Representative panels and gating strategy of experiments performed for obtaining data from Figure 5. (B) Fluorescence Minus One Control (FMO controls).

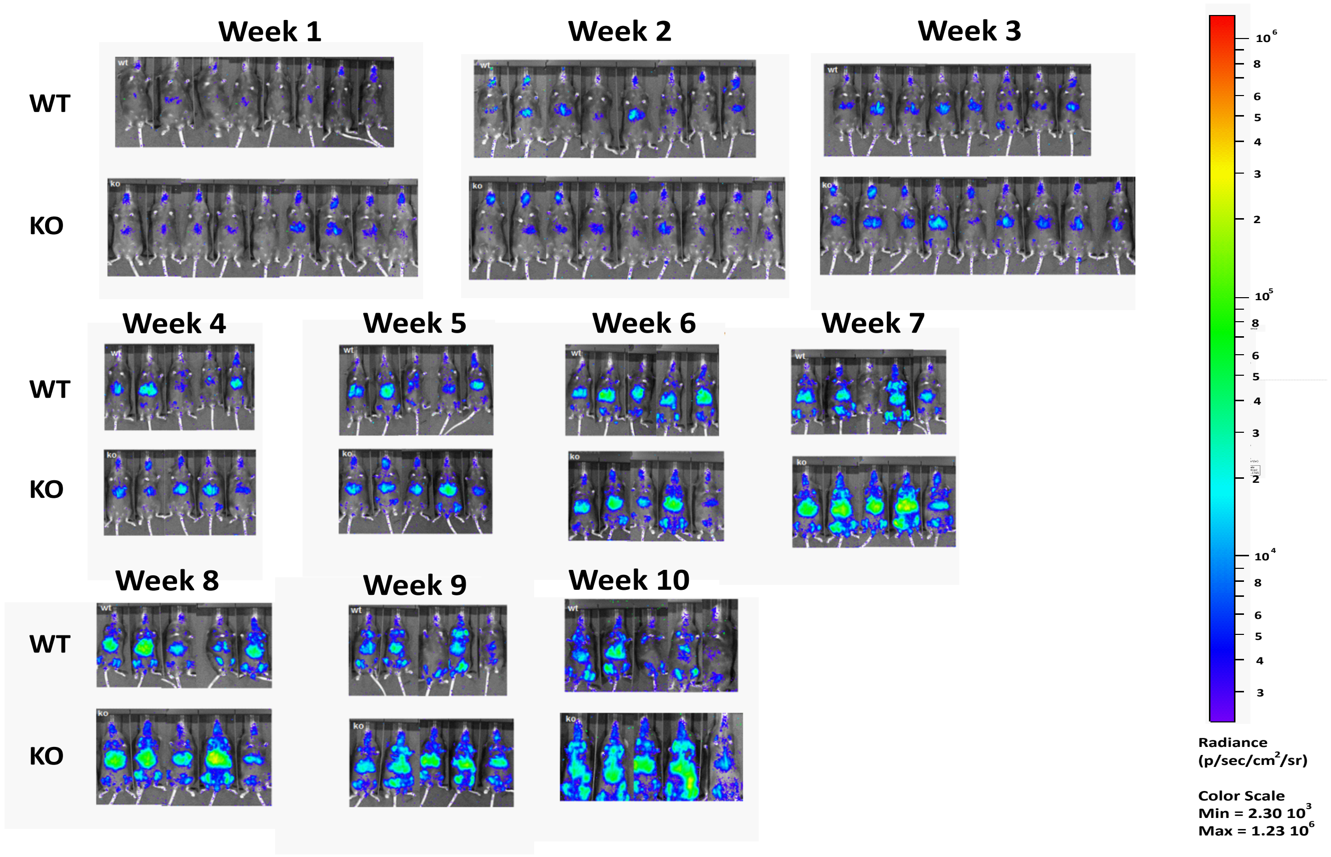

Week 1
Week 2
Week 3
Week 4
Week 5
Week 6
Week 7
Week 8
Week 9
Week 10
WT
KO
wt
ko
Radiance (p/sec/cm²/sr)
Color Scale
Min = 2.30 10³
Max = 1.23 10⁶

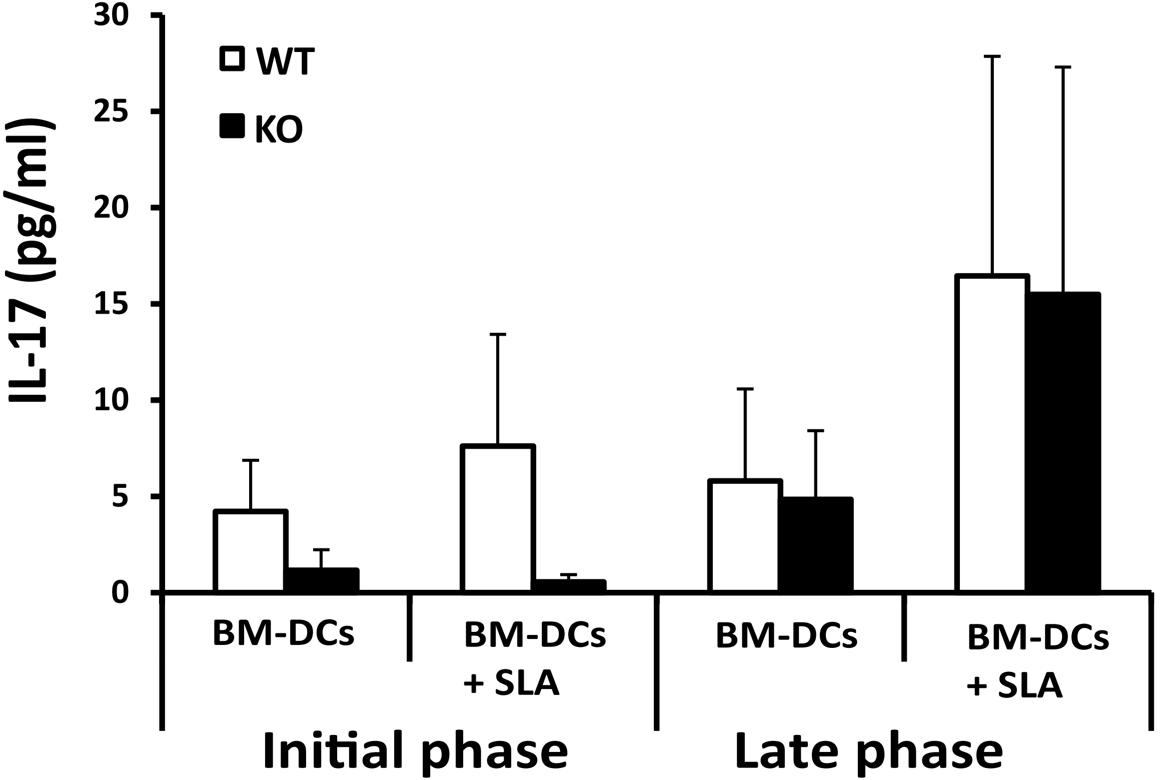
IL-17 (pg/ml)
30
25
20
15
10
5
0
WT
KO
BM-DCs
BM-DCs + SLA
BM-DCs
BM-DCs + SLA
Initial phase
Late phase

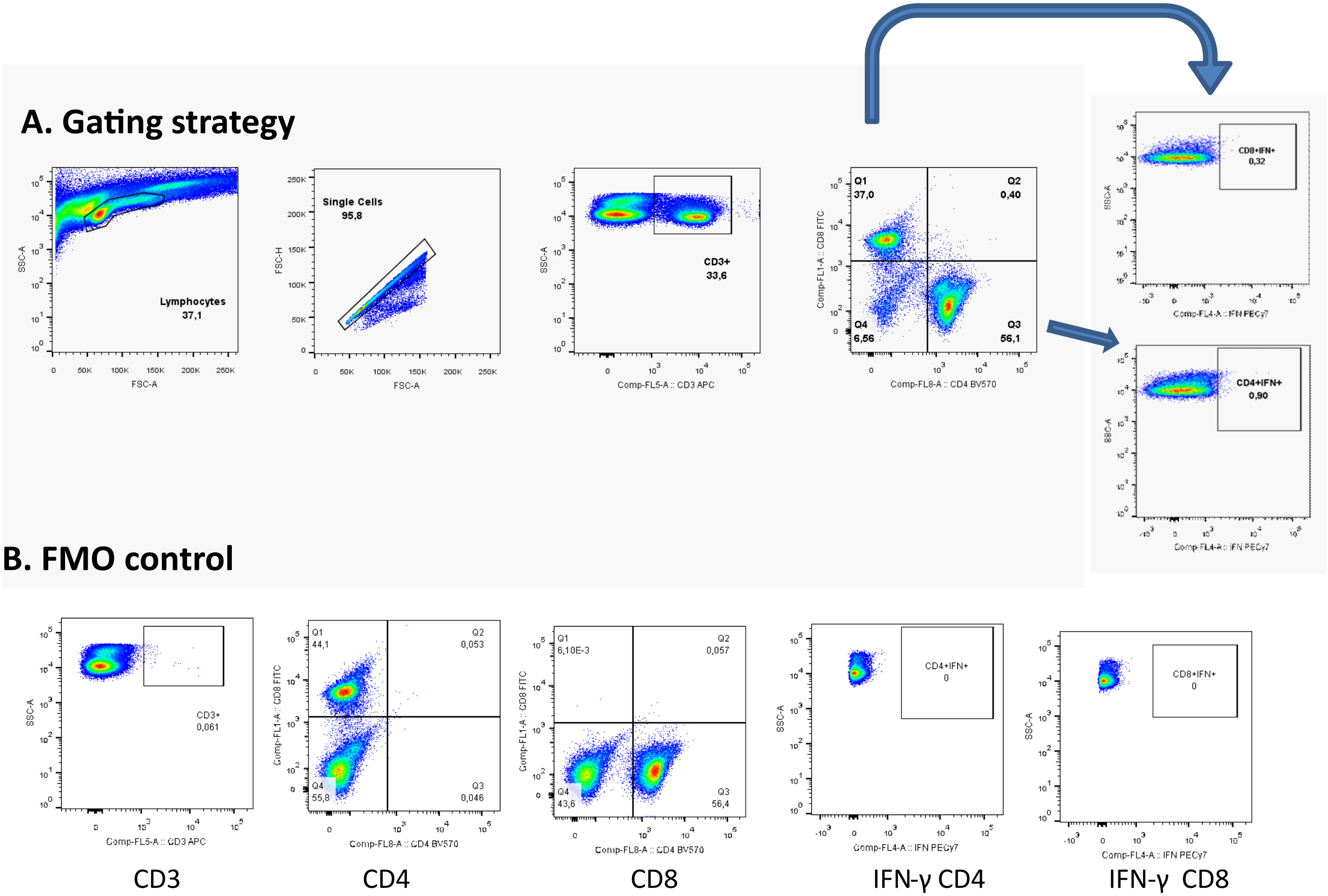

A. Gating strategy
Lymphocytes 37,1
SSC-A
FSC-A
Single Cells 95,8
FSC-H
CD3+ 33,6
Comp-FL5-A :: CD3 APC
Q1 37,0
Q2 0,40
Q3 56,1
Q4 6,56
Comp-FL1-A :: CD8 FITC
Comp-FL8-A :: CD4 BV570
CD8+IFN+ 0,32
CD4+IFN+ 0,90
Comp-FL4-A :: IFN PECy7
B. FMO control
CD3+ 0,061
Q1 44,1
Q2 0,053
Q3 0,046
Q4 55,8
Q1 6,10E-3
Q2 0,057
Q3 56,4
Q4 43,6
CD4+IFN+ 0
CD8+IFN+ 0
CD3
CD4
CD8
IFN-γ CD4
IFN-γ CD8
